# The Matron's Department

**Published:** 1905-09-23

**Authors:** 


### HOSPITAL ADMINISTRATION.

### CONSTRUCTION AND ECONOMICS.

### THE MATRON'S DEPARTMENT.

#### VI. THE NEW PROBATIONER.

The responsibilities of the matron with regard to her probationers begin long before they enter the hospital. The task of selecting them bears often very heavily on her. In some cases thousands, and in many cases hundreds, of applications are received yearly from intending nurses, and to secure the right women out of this crowd of applicants is a severe strain on the judgment.

It is astonishing, in view of this large field of choice, that so few hospitals require any preliminary knowledge from their probationers. Candidates are frequently selected many months before a vacancy occurs, and during this time might be wholesomely employed in preparing themselves for their future duties, but it is the exception for any course of study to be laid down for them.

At the London Hospital and at Guy's there are preliminary schools for accepted probationers, in which instruction is given to them for a few weeks before they are passed on into the wards. At St. Bartholomew's Hospital, after a preliminary examination to test general intelligence and education, the probationers are examined in elementary physiology, the composition of the air, the structure and use of thermometers, and the signs and terms commonly used in prescriptions. At the Leicester Infirmary applicants must pass a preliminary examination in elementary physiology and in sick-room cookery. At the Glasgow Royal Infirmary a three months' course of serious study, divided into two courses, is required from all candidates, who provide board and lodging at their own expense. The first course consists of twelve lectures and demonstrations on anatomy, twelve on physiology, and twelve on hygiene. Those who pass the examination at the conclusion of this course are admitted to the second course, consisting of twenty lectures and demonstrations on surgical cases, twenty on medical cases, and twenty practical lectures by the matron and housekeeper on ward work and cookery. Not till they have passed an examination in the subjects of the second course are they admitted as probationers. After entering the hospital the instruction is purely practical.

It is hardly likely that preliminary probationer schools will ever become universal. The expense is prohibitive, that at the London Hospital costing the charity £1,200 a year. Nor is it practicable in ordinary hospitals for the staff to arrange courses for outside students desirous of becoming probationers.

But it is both desirable and practicable that intending probationers should have a definite course of preparation marked out for them. From the table subjoined it will be seen how large a proportion of the entire staff necessarily consists of first-year probationers. The strain of the ward work arises in great measure out of the incapacity of new-comers. The labour of teaching them what they might with ease and satisfaction have learned before entering the hospital is a serious tax on the whole staff, from the medical officer—who tries to make them understand the circulation of the blood—to the harassed staff nurse, who must show them how to hold a broom.

In the first place, it is reasonable to expect intending probationers shall make themselves proficient in what is generally called "house-work." They ought to know how to make beds, dust rooms, sweep floors, light fires, polish brass, etc., all in the correct way, and to this they should add a practical knowledge of plain cookery. In many instances instruction of this nature is accessible to a girl in her own home. But where this is not the case, there are now many "schools of domestic management," where these elementary duties are properly taught. On this general knowledge special methods can be speedily grafted. A probationer who has practised herself in housework will see at a glance the orthodox method of making the beds, and with only a hint or two from her fellow-workers becomes a dexterous assistant. If she has practised the art of cooking, invalid cookery becomes merely a question of following a recipe. She should be required then to make herself familiar before she enters the hospital with the arts of keeping a house clean and cooking her own meals; and these are arts, it may be added, without which no woman's education is complete, whatever her sphere in life may be.

And, in the second place, the probationer should be urged to master at least the elements of physiology before she begins her training.

The Board of Education holds periodical examinations in London and most provincial towns on this subject, and excellent courses of instruction are given in connection with them. The standard of knowledge required for Human Physiology, Stage I., includes all that is usually taught to nurses in this connection, viz.:—

1. The form, position, and use of the bones constituting the skeleton, with a general idea as to the build of the body, as well as the boundaries and position of the contents of the various body cavities.

2. The structure and functions of the skin.

3. The structure and arrangement of the heart, chief blood-vessels, and the circulation, with a knowledge of the general composition, uses, and phenomena of blood. The microscopic appearance of human blood.

4. The structure and arrangement of the lungs, with a knowledge of the theory of respiration and the changes resulting therefrom in the blood and air.

5. The general structure and uses of the teeth, stomach, and intestines, including a knowledge of the processes of digestion and assimilation.

6. The position, general structure, and uses of the liver, spleen, pancreas, kidneys, and bladder.

7. The muscular system. The nature of joints. Animal heat.

8. The different kinds of sensations. The eye. The ear.

9. The nervous system.

The examination is purely elementary, and, after a reasonable period of study, should present no difficulty to candidates who have had a modern education and have passed, for instance, the seventh standard in the elementary schools.

It is believed that many intending probationers would find interest in advancing to the second stage. It is certain that they would derive very much benefit from attendance on a course of hygiene as laid down by the Board of Education. Lectures on physiology and hygiene are held in all large centres in connection with the Science and Art Department at South Kensington at very moderate terms and would, we believe, be eagerly attended by young women desiring to fit themselves for their future duties as nurses, if their attention were drawn by the hospital authorities to the advantage of some preliminary knowledge.

It will not rest with the matron to determine the regulations for admitting probationers. But her advice will have weight with the hospital authorities, and even without formal requirements she may do much to influence her accepted candidates to prepare themselves for their work. She will do well to impress upon them at the very least a preliminary course of early rising, for want of which many promising probationers have been known to collapse altogether after their first week.

| — | Number of First Year Probationers. | Number of Sisters. | Percentage of First Year Probationers in Total Staff. |
|---|---|---|---|
| Hospitals with Medical Schools. | | | |
| The London Hospital ... ... ... | 147 | 70 | 34 |
| The Middlesex ... ... .. ... | 57 | 10 | 41 |
| St. Mary's ... ... ... ... | 28 | 12 | 28 |
| University College ... ... ... | 36 | 12 | 45 |
| Charing Cross ... ... ... ... | 15 | 13 | 24 |
| The Royal Free... ... ... ... | 12 | 9 | 24 |
| Birmingham General ... ... ... | 24 | 15 | 23 |
| Sheffield Royal ... ... ... ... | 20 | 13 | 30 |
| Bristol General... ... ... ... | 45 | 9 | 51 |
| Addenbrooke's ... ... ... ... | 12 | 8 | 26 |
| Edinburgh Royal Infirmary ... ... | 80 | 33 | 35 |
| Glasgow Royal Infirmary ... ... | 30 | — | 19 |
| Glasgow Western Infirmary ... ... | 40 | 13 | 26 |
| Belfast Royal Victoria... ... ... | 11 | 9 | 16 |
| Hospitals without Medical Schools. | | | |
| The Seamen's ... ... ... ... | 18 | 6 | 39 |
| Great Northern Central ... ... | 11 | 9 | 25 |
| Royal Devon and Exeter ... ... | 24 | 9 | 30 |
| Norfolk and Norwich ... ... ... | 13 | 8 | 24 |
| Sunderland Infirmary ... ... ... | 10 | 7 | 23 |
| Hull Royal ... ... ... ... | 9 | 8 | 20 |
| Royal Berkshire, Reading ... ... | 12 | 8 | 25 |
| S. Devon and E. Cornwall ... ... | 9 | 10 | 20 |
| Taunton and Somerset ... ... | 9 | 6 | 14 |
| Perth County ... ... ... ... | 5 | 5 | 20 |
| Ayr County ... ... ... ... | 8 | 5 | 32 |

The above table shows the percentage of first year probationers among the staff in certain representative hospitals. The average percentage is 28 and this will give some idea of the importance to the hospital of transforming the unskilled probationers in the shortest possible period of time to trained assistants.

